# Genomic deletions of *MSH2* and *MLH1* in colorectal cancer families detected by a novel mutation detection approach

**DOI:** 10.1038/sj.bjc.6600565

**Published:** 2002-10-07

**Authors:** J J P Gille, F B L Hogervorst, G Pals, JTh Wijnen, R J van Schooten, C J Dommering, G A Meijer, M E Craanen, P M Nederlof, D de Jong, C J McElgunn, J P Schouten, F H Menko

**Affiliations:** Department of Clinical Genetics and Human Genetics, Cancer Family Clinic, VU University Medical Center, 1007 MB Amsterdam, the Netherlands; Department of Pathology, VU University Medical Center, 1007 MB Amsterdam, the Netherlands; Department of Gastroenterology, VU University Medical Center, 1007 MB Amsterdam, the Netherlands; Cancer Family Clinic and Department of Pathology, Netherlands Cancer Institute, 1066 CX Amsterdam, the Netherlands; Department of Human Genetics, Leiden University Medical Center, 2300 RC Leiden, the Netherlands; MRC-Holland, 1057 SN Amsterdam, the Netherlands

**Keywords:** mismatch-repair, HNPCC, genomic deletions, Muir–Torre syndrome

## Abstract

Hereditary non-polyposis colorectal cancer is an autosomal dominant condition due to germline mutations in DNA-mismatch-repair genes, in particular *MLH1*, *MSH2* and *MSH6*. Here we describe the application of a novel technique for the detection of genomic deletions in *MLH1* and *MSH2*. This method, called multiplex ligation-dependent probe amplification, is a quantitative multiplex PCR approach to determine the relative copy number of each *MLH1* and *MSH2* exon. Mutation screening of genes was performed in 126 colorectal cancer families selected on the basis of clinical criteria and in addition, for a subset of families, the presence of microsatellite instability (MSI-high) in tumours. Thirty-eight germline mutations were detected in 37 (29.4%) of these kindreds, 31 of which have a predicted pathogenic effect. Among families with MSI-high tumours 65.7% harboured germline gene defects. Genomic deletions accounted for 54.8% of the pathogenic mutations. A complete deletion of the *MLH1* gene was detected in two families. The multiplex ligation-dependent probe amplification approach is a rapid method for the detection of genomic deletions in *MLH1* and *MSH2*. In addition, it reveals alterations that might escape detection using conventional diagnostic techniques. Multiplex ligation-dependent probe amplification might be considered as an early step in the molecular diagnosis of hereditary non-polyposis colorectal cancer.

*British Journal of Cancer* (2002) **87**, 892–897. doi:10.1038/sj.bjc.6600565 www.bjcancer.com

© 2002 Cancer Research UK

## 

Hereditary non-polyposis colorectal cancer (HNPCC) is an autosomal dominant predisposition for early-onset colorectal cancer, endometrial cancer and other malignant tumours. Many HNPCC families fulfil the ‘Amsterdam criteria’ which require three colorectal cancer patients, vertical transmission and young age at diagnosis (Vasen *et al*, 1991). In about 50% of families that meet these criteria, germline mutations in one of the DNA-mismatch-repair (MMR) genes *MSH2* or *MLH1* are detected (Wijnen *et al*, 1997, 1998a). Virtually all colorectal tumours from *MSH2* or *MLH1* mutation carriers show microsatellite instability (MSI), which reflects the defect in DNA-mismatch-repair, and absence of expression of the MMR gene product involved. MSI is an important but not a specific marker of a germline MMR gene defect: the instability can also be due to acquired hypermethylation of *MLH1* (Raedle *et al*, 2001). A minority of HNPCC families has a *MSH6* defect or, exceptionally, a mutation in one of the other MMR genes (Liu *et al*, 2001). Tumours due to *MSH6* mutations may or may not show MSI (Berends *et al*, 2002). Germline mutations in MMR genes can lead to a variety of clinical presentations, including sporadic early-onset colorectal cancer and familial endometrial cancer (Farrington *et al*, 1998; Wijnen *et al*, 1999). Therefore, additional clinical criteria were developed which may indicate an MMR gene defect, in particular the Bethesda and Amsterdam II criteria (Rodriguez-Bigas *et al*, 1997; Vasen *et al*, 1999). Identification of a germline defect in colorectal cancer patients is crucial to establish the etiology of the disease and to direct clinical decision making for patients and family members.

At present, mutation detection of DNA-mismatch-repair genes is often performed by DGGE (Denaturing Gradient Gel Electrophoresis), DHPLC (Denaturing High Performance Liquid Chromatography), SSCA (Single Strand Conformation Analysis) and direct DNA sequencing. With these methods single base substitutions and small deletions and insertions can be detected. However, genomic deletions, i.e. deletions of one or more entire exons as well as duplications of exons will not be identified by these techniques. Detection of this class of mutations is important since they are an important cause of hereditary non-polyposis colorectal cancer (Wijnen *et al*, 1998b).

Conventionally, the main technique used for the detection of genomic deletions in MMR genes is Southern blotting (Wijnen *et al*, 1998b). However, Southern blot analysis has several drawbacks: the method is laborious and time-consuming and requires a relatively large amount of high-quality DNA. Therefore, alternative methods were developed based on (semi-) quantitative PCR (Charbonnier *et al*, 2000, 2002; Wang *et al*, 2002).

Here we describe the value of a new technique, called Multiplex Ligation-dependent Probe Amplification (MLPA), for determination of the relative copy numbers of DNA sequences, and application of this method for the analysis of genomic deletions in *MSH2* and *MLH1*.

## MATERIALS AND METHODS

We studied a cohort of colorectal cancer families investigated at the Cancer Family Clinics of the VU University Medical Center and the Netherlands Cancer Institute, Amsterdam, the Netherlands. The aim of this study was to assess the value of the MLPA test for the detection of genomic *MSH2-* or *MLH1-*deletions.

In 126 colorectal cancer families germline mutation analysis of *MSH2*, *MLH1* and/or *MSH6* was performed. *MSH1* and *MLH2* were analysed in 103 kindreds. *MSH6* was analysed in most families negative for *MSH2* and *MLH1*. In the other 23 families, all with MSI-stable or MSI-low tumours, only *MSH6* was examined.

The pedigree data either fulfilled the Amsterdam criteria I or II for HNPCC or less stringent criteria, usually one or several of the Bethesda criteria (‘suspected HNPCC’). Germline mutation screening of MMR genes was performed using DGGE or SSCA in combination with DHPLC for *MLH1* and *MSH2* and DGGE for *MSH6*. Investigation of MSI in tumours was performed according to standard procedures (Boland *et al*, 1998). Southern blotting of *MSH2* and *MLH1* was performed in one institution (VUMC) for families with MSI-high tumours and a previous negative screening for MMR gene defects. DNA samples from all 126 families were subjected to MLPA analysis. Informed consent was obtained from all patients who underwent DNA-based diagnosis.

**Figure 1:**
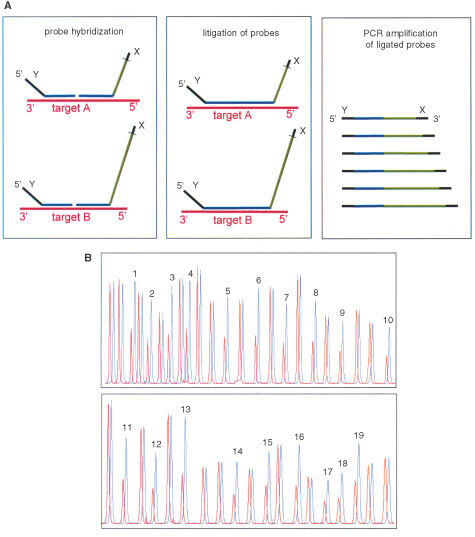
(**A**) Multiplex Ligation-dependent Probe Amplification (MLPA). Denatured genomic DNA (50–500 ng) is hybridised with a mixture of 42 probes. Each MLPA probe consists of two oligonucleotides. The two parts of each probe hybridise to adjacent target sequences and are ligated by a thermostable ligase. All probe ligation products are amplified simultaneously by PCR using a single primer pair. The amplification product of each probe has a unique length (130–472 bp). Amplification products are separated by capillary electrophoresis (ABI model 310 or ABI 3700). Relative amounts of probe amplification products reflect the relative copy number of target sequences. (**B**) Deletion of the entire *MLH1* gene detected by MLPA. Normalised MLPA peak pattern from the index patient of family C149 (red) and from control DNA (blue) plotted in one figure for easy comparison. *MLH1* peaks are labelled with their exon numbers. Unlabelled peaks represent *MSH2* exons and control genes.

In MLPA, illustrated in Figure 1A

, probes are used that consist of two oligonucleotides, both having a sequence complementary to a part of a target sequence at one site and a universal primer annealing sequence at the other site. Both hemi-probes can hybridize directly adjacent to each other to a target genomic sequence. If hybridized, the hemi-probes can be ligated and subsequently be amplified by PCR. Non-hybridized hemi-probes do not have to be removed since they can not be ligated and consequently will not be amplified. By using probes with amplification products of different lengths, multiple probes can be used in one reaction and be separated by gel electrophoresis for quantification (Schouten *et al*, 2002).

The MLPA test for *MSH2* and *MLH1* was obtained from MRC-Holland, Amsterdam, the Netherlands. The probe mix contains 16 exon probes for *MSH2*, 19 exon probes for *MLH1*, and seven control probes specific for DNA sequences outside the *MSH2* and *MLH1* genes. Details on probe sequences can be found on http://www.mrc-holland.com. All incubations were performed in a PCR machine with heated lid. Genomic DNA (50–500 ng) in 5 μl TE was denatured at 98°C for 5 min. Next, 3 μl probe mix was added and the mix was heated at 95°C for 1 min. and incubated at 60°C for 16 h (‘overnight’). Ligation was performed using heat-stable Ligase-65 enzyme at 54°C for 15 min, followed by ligase inactivation at 98°C for 2 min. Next, 10 μl ligation mix was added to 40 μl PCR buffer containing dNTPs, Taq polymerase and PCR primers (one unlabelled and one FAM-labelled primer). The reaction mixture was preheated at 95°C for 1 min. followed by 32 cycles of denaturation at 95°C for 30 s, annealing at 60°C for 30 s and extension at 72°C for 1 min. A final extension was performed at 72°C for 20 min. Fragment analysis was carried out on ABI model 310 or 3700 capillary sequencer (Applied Biosystems, Forster City, CA, USA) using TAMRA-500 or ROX-500 as size standards. A peak pattern of 42 peaks ranging in size from 130 to 472 nt is obtained.

Data analysis was performed using Genescan and Genotyper software (Applied Biosystems). For analysing a small series of samples, visual inspection of the peak pattern of a patient's sample superimposed over a peak pattern of a control is very suitable (see Figure 1B). For analysing large series of samples, peak areas were imported into Excel spreadsheets and peak fractions were calculated by dividing the peak area of a certain probe by the sum of peak areas of all control probes in a certain sample. Subsequently, this relative peak area of each probe was divided by the average relative peak area of this probe in control samples. In normal individuals this calculation will result in a value of 1.0 representing two copies of the target sequence in the sample.

## RESULTS

The yield of germline mutation analysis according to the clinical subgroup and the MSI status of tumours is given in Table 1

**Table 1:**
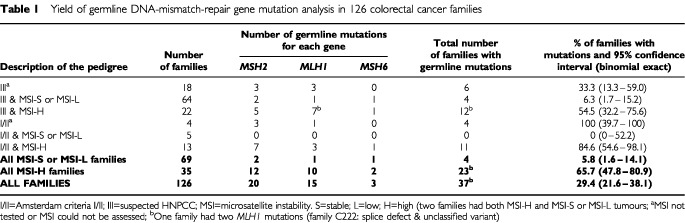
Yield of germline DNA-mismatch-repair gene mutation analysis in 126 colorectal cancer families

. We found germline mutations in 37 (29.4%) of the 126 colorectal cancer families. The characteristics of these families are summarised in Table 2

**Table 2:**
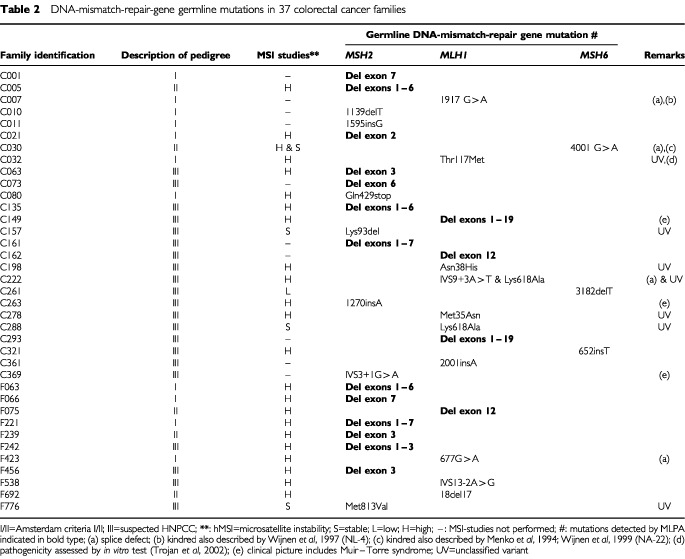
DNA-mismatch-repair-gene germline mutations in 37 colorectal cancer families

**Figure 2:**
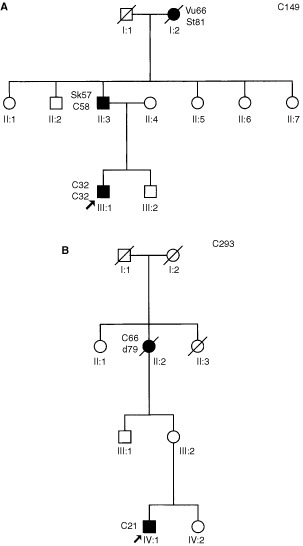
(**A**) Family C149. The index patient had a double colonic tumour at age 32, his father had colonic cancer preceded by skin tumours including Muir–Torre syndrome skin lesions (sebaceous gland adenoma, kerato-acanthoma, planocellular cancer). Patient 1-2 had primary cancers of the vulva and stomach. (**B**) Family C293. The index patient had colonic cancer at the age of 21 years. His unaffected mother had a tubular adenoma on colonoscopic screening, his maternal grandmother had late-onset colonic cancer. Family members I-2 (C149) and I-1 (C293) have a common ancestor.

. Thirty-eight germline mutations were found, including a double mutation in one kindred (family C222: a splice defect and an unclassified variant of *MLH1*), 20 in *MSH2*, 15 in *MLH1* and three in *MSH6*. Seven (six different) DNA alterations were of unknown clinical significance. Thirteen genomic deletions were identified in *MSH2* and four in *MLH1*, including a deletion of the entire *MLH1* gene in two families (Figure 1B). These two kindreds were found to have a common ancestor in the eighteenth century. The clinical picture is summarised in Figure 2

. All genomic deletions detected by Southern blotting were confirmed by MLPA.

## DISCUSSION

We found a high frequency of genomic deletions in *MSH2* and *MLH1* in a large cohort of colorectal cancer families. The new MLPA technique efficiently detected these deletions. In general, the yield of MMR gene mutation analysis in colorectal cancer families depends upon: (1) the selection of families based on clinical criteria, (2) the MSI status of tumours and/or immunohistochemical expression of MMR gene products, (3) the methods used for germline mutation analysis and (4) the interpretation of mutations as pathogenic *vs* non-pathogenic.

The families we studied include a wide range of clinical conditions with varying indications of HNPCC. They were classified into two main groups: those that fulfil the Amsterdam criteria (I or II) and other ‘suspected’ families. Families from the latter group generally satisfied one or more of the Bethesda criteria. It should be noted that the description of a given family according to the Amsterdam or Bethesda criteria does not always give the most complete clinical information. For example, Muir–Torre syndrome skin lesions were observed in several families from our study group and strongly indicate HNPCC; however, they are not mentioned in the above-mentioned criteria. In addition, many families seen in the clinic have a strong but unconfirmed family history of colorectal cancer whereas the Amsterdam and Bethesda criteria require histologically confirmed diagnoses.

In families that meet the stringent Amsterdam criteria, pathogenic *MSH2* or *MLH1* mutations have been detected in about 50% of kindreds (Wijnen *et al*, 1997). The same yield of about 50% is found for families with less clinical indications of HNPCC but which, in addition, have MSI-high tumours (Terdiman *et al*, 2001; Raedle *et al*, 2001). Among families that only fulfil clinical criteria, usually not as stringent as the Amsterdam criteria, mutations are detected in about 30% of kindreds (Wijnen *et al*, 1998a; Bapat *et al*, 1999; Lamberti *et al*, 1999; Syngal *et al*, 2000; Wahlberg *et al*, 2002). Depending on the specific subgroup which is tested, the frequency of mutations may be lower than 10% or higher than 90%.

Our mutation yield was 5.8% for families with MSI-S or MSI-L tumours and 65.7% for families with MSI-H tumours (Table 1). Our data support the notion that screening for MSI in tumours is an efficient prescreening tool for mutation analysis.

Since we included a technique for the detection of genomic deletions which revealed a high frequency of this class of mutations, one might argue that a frequency of non-deletion mutations of 20 out of 126 kindreds (15.9%) is lower than one would expect. Probably there are two main reasons for the low percentage of non-deletion mutations. First, we performed mutation analysis in a relatively large group of families which did not meet the Amsterdam criteria (I or II) and, in addition, had MSI-stable or MSI-low tumours. In 64 of these families we found only four (6.3%) germline mutations. These mutations include three unclassified variants in *MSH2* and *MSH1* and one pathogenic *MSH6* mutation. It has been observed before that deleterious *MSH6* mutations may be accompanied by MSI-low tumours (Berends *et al*, 2002). Second, in several families all affected individuals had died and, consequently, only at-risk family members were available for testing.

The methods we used for the analysis of point mutations were DGGE or SSCA in combination with DHPLC for *MSH2* and *MLH1* and DGGE for *MSH6*. These techniques are highly sensitive and specific for the detection of mutations in the coding regions of these genes (Wahlberg *et al*, 1999). Mutations in the promoter regions of *MSH2* and *MLH1* will not be detected by these methods; however, they seem to play a limited role (Shin *et al*, 2002). We found six different mutations in *MSH2* and *MLH1* categorised as unclassified variants; their clinical significance is uncertain. However, the Thr117Met variant in *MLH1* most probably has a pathogenic effect as demonstrated in a recently developed *in vitro* assay (Trojan *et al*, 2002).

In our cohort of colorectal cancer families more than 50% of the pathogenic mutations were genomic deletions. Evidently, genomic deletions in *MLH1* and *MSH2* are an important cause of HNPCC in Dutch colorectal cancer families. It has been reported previously that *MSH2* genomic deletions are frequent in this group. Haplotype analysis of the kindreds sharing the same deletions failed to show evidence of a founder effect (Wijnen *et al*, 1998b). Only two *MLH1* deletions had been reported at that time, one of which was a founder mutation that accounted for a large proportion of HNPCC kindreds in the Finnish population (Nyström-Lahti *et al*, 1995; Mauillon *et al*, 1996). In a recent study of German HNPCC kindreds a predominance of *MLH1* deletions was found (Wang *et al*, 2002). Recently, deletion mutations have also been demonstrated in series of European and US colorectal cancer kindreds, but at a much lower frequency than in our study group (Wijnen JTh, personal communication). We found 17 genomic deletions, 13 in *MSH2* and four in *MLH1*. These included at least nine different subtypes, which might imply that the high frequency of deletions in our study group is not caused by a strong founder effect. However, five deletions occurred in multiple kindreds and a common ancestor was demonstrated for the *MLH1* deletion. Future studies will clarify the frequency and background of genomic deletions in *MSH2* and *MLH1* in different populations.

A deletion of the entire *MLH1* gene has not been described before. In the two kindreds involved, the clinical expression included Muir–Torre syndrome and very-early-onset colonic cancer. A deletion of the complete *MSH2* gene in an HNPCC family was recently described by Wang *et al* (2002).

In summary, the MLPA technique is a fast and efficient test for the detection of genomic deletions in MMR genes. In contrast with the PCR- based techniques described by Charbonnier *et al* (2002) and Wang *et al* (2002) deletion screening of *MSH2* and *MLH1* by MLPA can be performed in a single procedure. Compared with the MAPH method developed recently (Armour *et al*, 2000) MLPA is easier to perform. The technique allows the detection of deletions that might escape detection using Southern blot analysis. In particular, interpretation of Southern blotting results may be difficult in case of the deletion of an entire gene.

Clearly, screening for genomic deletions in *MSH2* and *MLH1* is an essential procedure for the molecular diagnosis of HNPCC. Due to the advantages of the MLPA technique, screening for these deletions might be considered as an early step in MMR gene mutation analysis.
